# Contribution of the periosteum to mandibular distraction

**DOI:** 10.1371/journal.pone.0199116

**Published:** 2018-06-28

**Authors:** Alexandre Debelmas, Arnaud Picard, Natacha Kadlub, Jean Boisson

**Affiliations:** 1 APHP, Necker Enfant Malades, Unit of Maxillofacial Surgery and Plastic Surgery, Paris, France; 2 IMSIA, ENSTA Paris-Tech, Department of Mechanical Engineering, Palaiseau, France; 3 Paris Descartes University, Paris, France; University of Zaragoza, SPAIN

## Abstract

Mandibular distraction is a surgical process that progressively lengthens bone. To improve the distraction procedure and devices, the load of distraction and the mechanical strain of soft tissues during the process must be determined. We tested the assumption that it could be the periosteum primarily opposing distraction. Therefore we assessed the mechanical properties of the human mandibular periosteum and compared the stress-strain data with the torque measured on the activator during a cadaveric mandibular distraction. A 20 mm horizontal mandibular distraction was performed in 7 cadavers using standard distractors. Torque was measured with a torquemeter placed on the activation rods of the devices, providing a load (*L*_*t*_) for each millimeter of distraction. In parallel, 18 periosteum samples were harvested from 9 cadaver mandibles. Uniaxial tensile tests were performed on the specimens and an estimated load (*L*_*c*_) was calculated using periosteal stress-strain data and mandibular dimensions. During the distraction process, we observed an increase of the load *L*_*t*_ from 11.6 to 50.6 N. The periosteum exhibited a nonlinear viscoelastic stress-strain relationship, typical of biological tissues composed of collagen and elastin. The median *L*_*c*_ and *L*_*t*_ were not significantly different for the first millimeter of distraction. We demonstrated the periosteum is primarily responsible for opposing the distraction load.

## Introduction

Distraction osteogenesis (DO) is a surgical procedure consisting of the progressive lengthening of a bone segment (1 mm/day). The technique requires the implantation of a distractor device, and daily activation is responsible for the lengthening of the bone. The use of distraction is widespread in the craniofacial area for the treatment of congenital malformations or acquired large bone defects [1]. Despite the technical improvements that lead to miniaturized intraoral distractors [2, 3], their activation still requires a transmucosal or transcutaneous rod to rotate the endless screw. This activation rod may be responsible for multiple adverse events and discomfort [4]. To overcome these issues, new generations of distractor devices such as automated distractors or distractors with distant activation have been proposed, but are currently not suitable for human use [5, 6]. In a previous study, we demonstrated the feasibility of distant activation with a magnetically activated device for mandibular distraction [7]. Despite the increasing use of DO and the development of new mechanisms in the field of DO, the role of the surrounding soft tissues, mainly the periosteum, and their participation in the mechanical load opposing the distraction vector are not understood. Forces involved in the process are also not precisely determined and they are essential to develop new generation distractor devices [5, 7].

The periosteum is a bilayered soft tissue surrounding bone [8]. The outer fibrous layer contains primarily collagen and elastin fibres, whereas the inner layer has a higher cell density, primarily of osteoblasts and periosteum-derived mesenchymal stem cells (PDCs). The periosteum is anchored to the bone by Sharpey’s fibres, strong fibres with a high collagen content [8]. It exhibits the properties of a viscoelastic anisotropic material [9–11]. Its behaviour during a uniaxial tensile test has been characterized by a nonlinear and partially reversible stress-strain relationship, which varies according to the orientation of its fibres. The periosteum stress-strain curve has the same aspect as other connective tissues composed of collagen and elastin with an initial toe region with a low elastic modulus, followed by a heel (transition region) and a stiffer linear phase (high elastic modulus) before rupture. Moreover, detachment of the periosteum from its bone support results in natural shrinkage along the long and short axes. Hence, in vivo, the periosteum is pre-stressed on the bone [9, 10, 12, 13]. These properties are consistently observed but vary across species and according to the anatomical location [9–15]. Despite its known role in many bone regeneration aspects [8, 14], to our knowledge, no study of the biomechanics of the human mandibular periosteum has been conducted, and none have incorporated it into a mandibular distraction process (distraction may be compared to a uniaxial traction test).

We hypothesized that the load opposing the distraction process during its firsts millimeters—before the callus formation—is provided by the periosteum. In the first part of the study, we measured the torque at the endless screw of the devices during a mandibular distraction to determine the global load of distraction. As we assumed that distraction is similar to a uniaxial traction test, the second part explored the mechanical properties of the human mandibular periosteum, especially the stress generated through uniaxial elongation to determine the periosteal load of distraction. Finally, we compared the torque-based load of distraction to the periosteal stress-strain-based load to test our main hypothesis and to provide the first elements for a distraction analytic model.

## Materials and methods

From January to June 2017, 9 human cadavers (3 males and 6 females numbered from I to IX) were dissected. These cadavers were provided by the Ecole de Chirurgie, Agence Générale des Equipements et Produits de Santé-AGEPS, Assistance Publique Hopitaux de Paris-APHP, APHP. Permission to perform cadaveric study on cadaveric specimens was obtained from the institutional review board (Ecole de Chirurgie, AGEPS, APHP). All the cadaveric subjects had given their consent for the use of their body for medical research. Ages at death ranged from 75 to 97 years. Lengths of cold preservation (at -18°C) extended from 13 to 460 days. A complete 20 mm horizontal distraction with torque assessment was performed on 7 cadavers (III to IX). For each distraction a different distractor device was used. Eighteen periosteum samples (2 per left mandibular corpus) were harvested.

### A. Assessment of the load of distraction: *L*_*t*_ (*n* = 7 cadavers)

The torque-based load *L*_*t*_ was obtained following a two-stage procedure: all the distractors were calibrated prior to the cadaveric experiments in order to determine their specific torque-load relationship, and then the torque was measured for each millimeter at the activation rod during standard 20 mm right horizontal distractions.

#### Distractor calibration and *L*_*t*_ acquisition

For a given load exerted on the two plates of the distractor, the torque required to activate the distraction (i.e. to depart the two plates) depends on the screw-thread size and the friction inside the endless screw. To discard the influence of friction, all distractors were calibrated before cadaveric experiments for loads ranging from 0 to 60 N from the first to the twentieth millimeters of distraction, using a custom-made apparatus (See S1 Text). Each device required four clockwise turns of the endless screw to achieve a 1 mm displacement. For each mm, the torque (in mN.m) on the activation rod during 5 s acquisitions was measured with a torque meter (Center Easy TT, Andilog Technologies, Marseille Vitrolles, France) at a sampling rate of 200 Hz. The torque values were averaged, providing a mean torque for each millimeter and for each load tested. This device-specific torque-load relationship provided an estimation of the load on the plate of the distractor for a given torque.

#### Cadaveric torque measurements

In this section, cadaveric distraction followed typical patient workflow [16]. A mandibular distraction was performed on the right horizontal branch of the mandible. Through an intra-oral approach, the mucosa and periosteum were incised longitudinally at the upper part of the mandible. Then, the periosteum was lifted 40 mm on the vestibular side and 10 mm on the lingual side of the mandible, then a complete vertical osteotomy of the mandibular corpus was performed. A 20 mm mandibular distractor device (Medicon eG, Tuttlingen, Germany) was subperiosteally implanted and fixed with four 5 mm titanium screws. The mucosa and periosteum were sutured and the endless screws were inserted through the mucosa (S2A Fig). The torque was measured on the activation rods during the entire distraction (20mm) with the same torquemeter, and the mean torque values for each millimeter were recorded using the same protocol as described above. Finally, the load of distraction *L*_*t*_ was determined using a linear interpolation between each point of the device-specific torque-load curves.

### B. Periosteal mechanical behaviour: *L*_*c*_

The primary objective of this study was to evaluate the mechanical role of the periosteum involved during distraction. As we assumed that a mandibular corpus distraction would stretch the periosteum and the other surrounding soft tissues along a single, anteroposterior axis, the next phase of our study was to characterize the periosteum behavior during a uniaxial tensile test.

#### Periosteum sample harvest and histological assessment

For each subject, the vestibular and lingual periosteum were harvested from the left mandibular corpus using a transmandibular approach (S2B Fig). Templates of 40 mm per 10 mm were drawn on the surface of the mandibular corpus (both lingual and vestibular sides) in an anteroposterior direction consistent with the horizontal mandibular distraction direction. Dimensions were set before harvesting the tissue to compensate for natural shrinkage. The periosteum is first detached from the overlying soft tissue. Then, the periosteum was cut following the drawings; slices were collected using a sharp elevator and immediately stored in saline solution. Specimens were removed from the saline solution a few hours after harvest and suspended 10 minutes in ambient air in a vertical position to drain the excess water. The objectives of this procedure were to allow complete relaxation, to avoid desiccation, and to limit the artefact due to tissue swelling.

Separate samples (5 by 10 mm, see S2B Fig) of each periosteal specimen were harvested at the posterior end of the tested specimens, fixed in formol and embedded in paraffin. Sections 4 *μ*m thick were cut from each paraffin block. Staining with haematoxylin/eosin/saffron (HES) was automated using a Leica Autostainer (Leica Biosystems GmbH, Nussloch, Germany). The sections were mounted in synthetic resin (WVR International, Radnor, PA, USA). The mean thickness (*h*) of the periosteum was measured on cross-section slides (averaged on 5 random thickness measures for each samples) using Image J version 1.48 (public domain software, National Institutes of Health, Bethesda, MD, USA).

### C. Parameters and tensile protocol

#### Tensile tests and protocol

**Fig 1:**
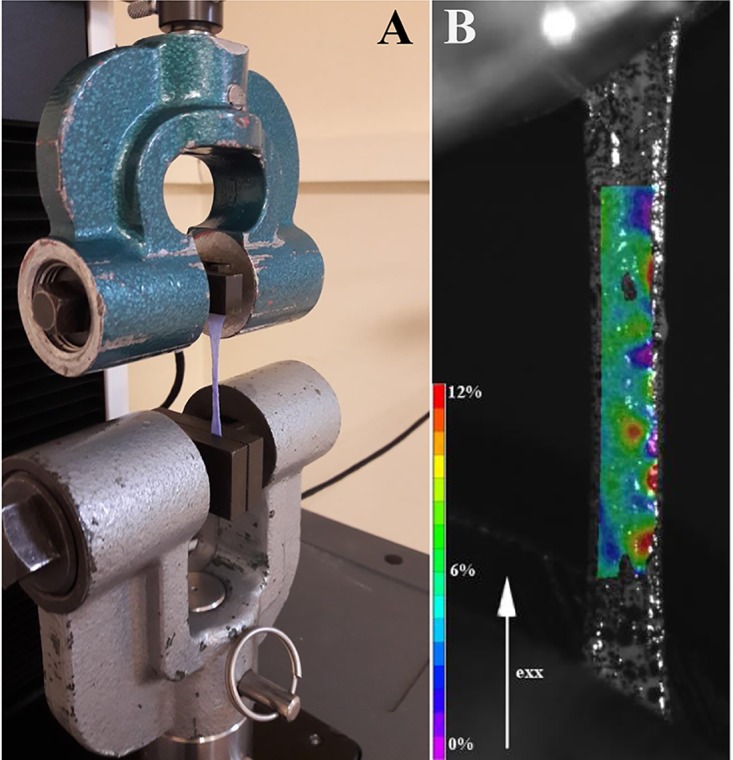
A: Picture of the tensile test apparatus. The elongation procedure were recorded with a high resolutionvideo camera. The speckled periosteal sample can be seen between the grips of the machine. B: Example of axial strain homogeneity on sample n°12. Tensile tests were recorded, and Lagrange deformation fields were calculated using digital image correlation. The Vic-2D parameters used to perform this analysis included a step of 3 pixels, Gaussian subset weights, optimized 8-tap interpolation, normalized square differences, and incremental correlation. The consistency threshold was set at 0.05 (maximum margin), the maximum confidence margin was 0.05. In this figure, the axial strain (*e*_*xx*_) is displayed with a colour scale.

A tensile test protocol was determined in order to simulate the periosteum condition during distraction. Tensile tests were performed with a uniaxial elongation machine (3342 Single Column, Instron Corp., Illinois Tool Works Inc., Glenview, IL, USA) at room temperature (18-20°C). Each periosteal sample was moistened every 3 min during the experiment using an atomizer (see Fig 1A). Periosteum slice ends were secured in two grips connected to the elongation device. The distance between the grips is controlled at a precision of 0.01 mm. The bottom jaw was fixed on the machines base; the upper jaw could move at a specified speed in the vertical direction and was fixed to a static load cell with a 100 N capacity (2519 series, Instron Corp., Illinois Tool Works Inc.).

Stress is defined as a load normalized on a surface. Hence, we have:

$$\begin{array}{c} \sigma =\frac{L}{w\times h} \end{array}$$ (1)

where *σ* is the axial stress in megapascals (MPa), *L* is the load in newtons (N) recorded by the load cell (see above for the load cell characteristics), and *w* × *h* is the initial cross-sectional area of the specimen (in m^2^) perpendicular to the direction of stretch; *h* is the thickness of the periosteum measured on histology (see above); *w* is the initial width of periosteum slice measured on the bone.

Strain is defined as:

$$\begin{array}{c} \varepsilon_{g}=\frac{\Delta l}{l} \end{array}$$ (2)

where Δ*l* is the variation of distance between the two grips measured during the tensile test and *l* is the initial distance between the grips.

Periosteal test specimens were loaded in tension at a rate of 0.25 mm/s until 15% deformation, *ε*_*g*_ = 0.15 (Phase 1), and relaxed 300 s (phase 2). The traction rate was set at 0.25 mm/s, which approximately corresponds to the distraction speed in vivo. The duration of relaxation was arbitrarily set to 300 s, as a 24-hr relaxation (as in a surgical distraction) was impossible to conduct under experimental conditions.

The tensile test method was elaborated using Bluehill 3 software (Instron, Illinois Tool Works Inc.).

#### Strain homogeneity

To demonstrate the homogeneity of the deformation during a uniaxial tensile test, strain in the central region of the 5 samples was measured using digital image correlation (DIC). Samples were speckled using India ink, and periosteal elongation was recorded using a 5-megapixel resolution video camera (GO-5000M-PMCL, JAI, Copenhagen, Denmark). Images were processed using Vic-2D version 6.0.6 digital image correlation software (Correlated Solutions, Inc., Irmo, SC, USA) to compute the Lagrange deformation fields of the samples (e.g. Fig 1B). The measured strain in the central region is linear with *ε*/*ε*_*g*_ = *k*. Therefore, we corrected the strain *ε*_*g*_ by this coefficient to determine the strain *ε* of the sample homogeneous region. For the samples where the DIC was not available, we have corrected *ε*_*g*_ by the average coefficient $\bar{k}$ calculated on the other experiments. In the following, we only consider the strain of the homogeneous region *ε* ($\varepsilon =\varepsilon_{g}\bar{k}$).

#### Stress-strain and relaxation data

The periosteum stress-stain curve presents two linear regions with a low and high modulus (toe and steep regions, respectively). The toe and steep regions of the stress-strain curves of phase 1 were fitted using two linear functions. The elastic moduli, *E*_*toe*_ and *E*_*steep*_ (in MPa), are the slopes of the fits:

$$\text{Phase}\;\text{1}:\{\begin{array}{ll} \sigma_{toe} & =E_{toe}\times \varepsilon \\ \sigma_{steep} & =E_{steep}\times \left(\varepsilon -\varepsilon_{0}\right)+\sigma_{0}^{steep} \end{array}$$ (3)

where *ε*_0_ and $\sigma_{0}^{steep}$ are respectively the strain and stress values of the intersection point of the two linear fits.

For phase 2, the stress over time relation *σ*(*ε*, *t*), normalized by the stress at the beginning of the relaxation *σ*_*max*_, was fitted with the bi-exponential function

$$\text{Phase}\;\text{2}:\{\sigma \left(\varepsilon ,t\right)/\sigma_{max}=A\left(e^{-t/\tau_{1}}+e^{-t/\tau_{2}}\right)+\sigma_{\infty },$$ (4)

providing two characteristic times, *τ*_1_ and *τ*_2_, specific to each periosteal sample [17]. *σ*_∞_ is the residual stress over *σ*_*max*_.

#### Stress-strain-based load estimation

The first step was to determine the periosteum stress and strain on the bone (*σ*_0_ and *ε*_0_, respectively). According to Bertram et al., this physiological state corresponds to the transition point between the toe and steep regions of the stress-strain curve [12]. This observation is supported by studies conducted on other collagen tissues such as tendons [18]. Hence, we determined *ε*_0_ as the intersection point of the two linear curves fitting the toe and steep regions. *σ*_0_ is the value of the experimental stress-strain curve at *ε*_0_.

During experimental cadaveric distraction, the periosteum was lifted approximately 40 mm on the vestibular side and 10 mm on the lingual side. Therefore, assuming that the sharpey fibers clamped the periosteum on the bone everywhere except where the operator lifted it, we assessed that 1 mm of distraction corresponded to 2.5% and 10% strain for the vestibular ($\varepsilon_{f}^{v}$) and lingual ($\varepsilon_{f}^{l}$) sides. Accordingly the strain on each side of the mandibule is:

$$\begin{array}{c} \begin{array}{cccc} & \varepsilon_{f}^{v}-\varepsilon_{0} & & =0.025\\ & \varepsilon_{f}^{l}-\varepsilon_{0} & & =0.1 \end{array} \end{array}$$ (5)

for the vestibular and lingual sides, respectively.

Using the fit of the steep region of phase 1, we evaluated stresses $\sigma_{f}^{v}$, $\sigma_{f}^{l}$ on the vestibular and lingual side of the mandibule as:

$$\begin{array}{c} \begin{array}{cccc} & \sigma_{f}^{v} & & =E_{steep}\times \left(\varepsilon_{f}^{v}-\varepsilon_{0}\right)+\sigma_{0}^{steep}\\ & \sigma_{f}^{l} & & =E_{steep}\times \left(\varepsilon_{f}^{l}-\varepsilon_{0}\right)+\sigma_{0}^{steep} \end{array} \end{array}$$ (6)

To approximate the load generated by the periosteum during a distraction, we calculated a stress-strain-based load *L*_*c*_ for each cadaver.

$$\begin{array}{c} L_{c}=\sigma_{f}^{l}\times w^{l}\times h^{l}+\sigma_{f}^{v}\times w^{v}\times h^{v} \end{array}$$ (7)

where $\sigma_{f}^{l}$ and $\sigma_{f}^{v}$ are the stress measured at the *ε*_*f*_ strain for periosteal samples harvested on the lingual and vestibular sides of the mandible, respectively. We assumed a 60 mm perimeter for a complete mandibular corpus, in which the lingual and vestibular sides accounted for half of this perimeter (30 mm). Then, we defined *w*^*l*^ and *w*^*v*^ as the lingual and vestibular parts of the mandibular perimeter, respectively. *h*^*l*^ and *h*^*v*^ are the thickness of the lingual and vestibular periosteal slices (histological measurements), respectively.

#### *L*_*c*_-*L*_*t*_ comparison

The stress-strain-based estimated load *L*_*c*_ was compared to the torque-based load *L*_*t*_ for the first millimeter of distraction.

#### Data processing and statistical analysis

All curves were fitted using Matlab (MathWorks, Inc., Natick, MA, USA). Statistical analyses were conducted using SPSS Statistics for Windows, Version 23.0 (IBM Corp., Armonk, NY, USA). Significance was set at *p* < 0.05. Continuous variables were tested for normality using a Wilks-Shapiro test. The ones with normal distribution were displayed as the mean and standard error of the mean (SEM) and compared using a t-test. Non-parametric variables were displayed as the median and interquartile range (IQR) and compared using a Wilcoxon ranks test.

## Results

The mean thickness was *h* = 0.23 mm (the interquartile range, *IQR* = 0.11 mm, Table 1). The coefficient *k* are constant along the whole tensile test (for all strains). The values are for vestibular samples $k_{VI}^{v}=0.78$, $k_{VII}^{v}=0.64$, and the coefficients are for lingual samples $k_{VI}^{l}=0.68$, $k_{VII}^{l}=0.50$, $k_{VIII}^{l}=0.81$ $\bar{k}=0.68$.

**Table 1:** Thickness value of the periosteum measured on histological cross-section slides (averaged on 5 random thickness measures for each samples).

Cad.	I	II	III	IV	V	VI	VII	VIII	IX
*h*_*v*_	0.281	0.317	0.215	0.296	0.208	0.416	0.277	0.290	0.308
*h*_*l*_	0.167	0.179	0.140	0.243	0.136	0.187	0.185	0.139	0.176

### A. Load of distraction assessment: *L*_*t*_

#### Torque-based load estimates *L*_*t*_

To cope with outliers in the series of torque measurements, and to compare these values with stress-strain-based loads (*L*_*c*_), *L*_*t*_ was displayed as the median and IQR of each cadaver.

**Fig 2:**
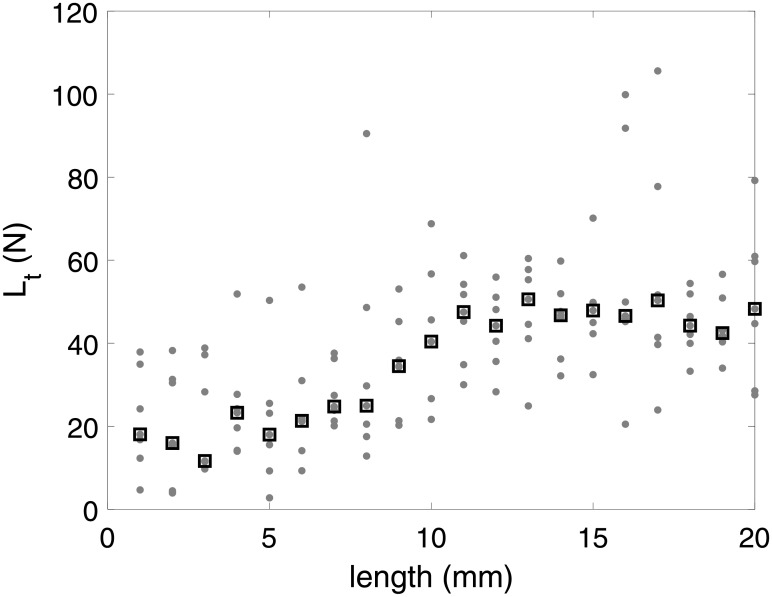
The grey points (⋅) corresponds to all *L*_*t*_ for all cadavers plotted against the length of the distraction during a 20 mm distraction. The black squares (□) are the medians of the load estimates calculated for each millimeter.

The torque-based load *L*_*t*_ was calculated for each millimeter of distraction in each cadaveric experiment (Fig 2). We observed a steady increase of *L*_*t*_ through the distraction process, with median loads ranging from 11.6 N to 50.6 N.

### B. Periosteal mechanical behaviour: *L*_*c*_

#### Stress-strain relationship and relaxation

**Fig 3:**
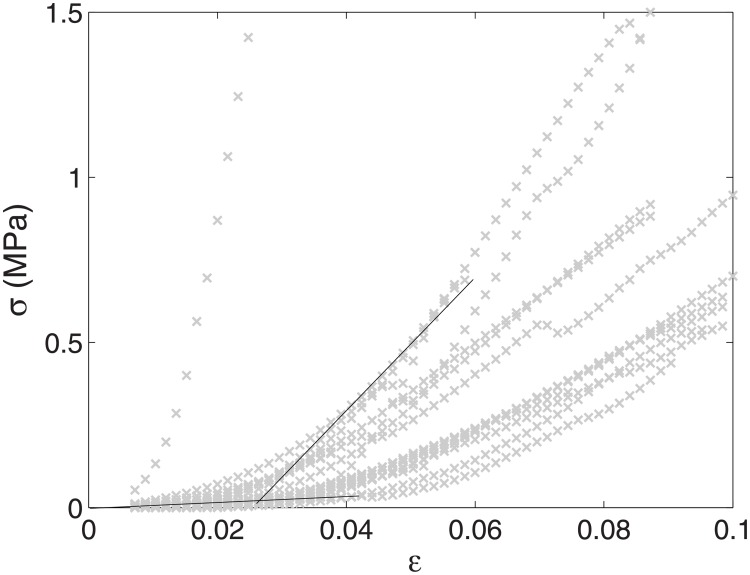
Phase 1 stress-strain curves. The axial stress *σ* (in MPa) is displayed against the axial strain *ε*. Dark lines represent the linear fits of *E*_*toe*_ and *E*_*steep*_ for sample *n*°12. We observed typical non-linear stress-strain curves with a toe region, a heel, and a linear steeper phase. Curves of all samples are represented with grey ×.

The digital image correlation showed homogeneous strains in the central region of the periosteum samples during tensile tests (Fig 1B). A typical non-linear stress-strain relationship was observed, with an initial toe region followed by a heel and a linear steep region (Fig 3). For phase 1, the median *E*_*toe*_ = 0.3 MPa (*IQR* = 0.6 MPa), while the median *E*_*steep*_ = 10.8 MPa (*IQR* = 10.0 MPa).

Regarding the transition region of the stress-strain curve, we measured a median *ε*_0_ = 0.032 (IQR = 0.01). The corresponding median stress is *σ*_0_ = 0.043 MPa (*IQR* = 0.031 MPa).

Parameters of phases 1, 2 for all the specimens are displayed in Table 2.

**Table 2:** Moduli of the steep linear region of the stress-strain curves in phase 1 (*E*_*steep*_), *ε*_0_ strains, *σ*_0_ pre-stresses, *τ*_1_ and *τ*_2_ characteristic times, stress-strain and torque-based loads (*L*_*c*_ and *L*_*t*_) are displayed for each periosteal sample and the corresponding cadaver. Moduli and pre-stress values are in megapascals (MPa). Characteristic times are in seconds (s). *L*_*c*_ and *L*_*t*_ loads are in newtons (N). Displayed *L*_*t*_ values are for the first millimeter of distraction (*s* = 1 mm). Loads were compared using a Wilcoxon signed-ranks test for paired samples. SEM: standard error of the mean IQR: interquartile range ⋆: calculated for cadaver III to IX. †: *L*_*c*_-*L*_*t*_ comparison.

Cad.	loc	*E*_*steep*_	*ε*_0_	*σ*_0_	*τ*_1_	*τ*_2_	*L*_*c*_	*L*_*t*_
I	v	8.1	0.033	0.018	127.8	9.6	7.3	n/a
	l	9.6	0.034	0.033	122.1	10.1		
II	v	7.7	0.030	0.035	122.8	8.4	7.0	n/a
	l	9.2	0.033	0.031	99.4	8.4		
III	v	29.7	0.037	0.244	388.5	68.1	13.3	34.9
	l	10.2	0.052	0.065	107.9	8.7		
IV	v	107.1	0.012	0.216	74.0	6.1	42.4	37.9
	l	24.9	0.036	0.049	99.3	6.3		
V	v	12.9	0.031	0.089	75.4	5.5	6.3	16.8
	l	9.6	0.021	0.040	107.2	9.3		
VI	v	27.1	0.035	0.119	126.5	7.5	20.4	18.0
	l	20.6	0.027	0.046	134.8	11.6		
VII	v	13.7	0.024	0.036	62.8	6.6	11.6	4.7
	l	15.6	0.029	0.038	92.4	7.7		
VIII	v	10.3	0.043	0.030	100.6	5.7	4.8	12.3
	l	4.2	0.025	0.025	143.8	9.7		
IX	v	8.0	0.021	0.052	105.1	8.9	8.4	24.2
	l	11.2	0.041	0.061	128.7	10.5		
Mean					123.3	11.6		
SEM					21.2	16.1		
Median		10.8	0.032	0.043			11.6^⋆^	18.0
IQR		10.0	0.010	0.031			11.8^⋆^	15.0
p							0.22^†^	

**Fig 4:**
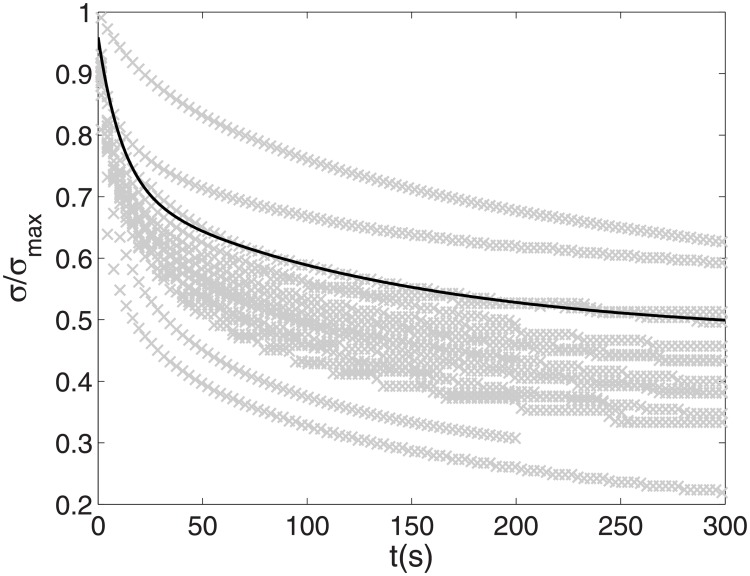
Relaxation curves. All samples are represented (grey points); the dark line represents the bi-exponential fit for sample *n*°12. The axial stress *σ* (in MPa), normalized with the maximum axial stress of the sample *σ*_*max*_ obtained at the end of phase 1 is displayed against time (in s). The following function was used to fit the relaxation curves: *R*^2^ > 0.983. It has to be noted that one sample relaxation stopped at 200 s for technical problem. We assume that the relaxation was long enough to perform a bi-exponentional fit.

We fitted the relaxation curves with the bi-exponential function with *R*^2^ superior to 0.98, giving a mean *τ*_1_ of 123.3 s (2*SEM* = 21.2 s), a mean *τ*_2_ of 11.6 s (2*SEM* = 16.1 s), and an average *σ*_∞_ of 0.4 (2*SEM* = 0.01). Relaxation curves and their parameters are displayed in Fig 4.

#### Stress-strain-based load estimation *L*_*c*_

For this part, we assumed that a non-surgically detached periosteum is still attached to the bone through the Sharpey fibers. We observed a median load *L*_*c*_ = 11.6 MPa (*IQR* = 11.8 MPa).

### C. *L*_*t*_-*L*_*c*_ comparison

The median *L*_*c*_ accounted for more than 64% of the median *L*_*t*_(*s*) for *s* = 1 mm. No statistical difference was found between *L*_*c*_ and *L*_*t*_ (*p* = 0.22). The results are summarized in Table 2.

## Discussion

In this study, we assumed that a uniaxial tensile test and distraction would generate similar periosteal mechanical stress. Our results showed that the periosteum is the primary tissue contributing to the load-opposing distraction at the beginning of distraction activation.

### Material behaviour (*σ*(*ε*, *t*))

The stress-strain relationship of the human mandibular periosteum is typical of biological connective tissues. Indeed, we observed nonlinear J-shaped stress-strain curves, stress relaxation at constant strain. [18]. Most of these features have already been described in the literature on the periosteum in other anatomical locations or species [8]. For instance, Bertram et al. [12] found a mean modulus of 229.5 MPa (±89.6, *n* = 72), when testing leghorn chicks tibiotarsal periosteum in an axial direction. They also reported a change in periosteal tensile properties over the course of growth, namely an increase in periosteal stiffness in the early period, followed by a decrease in the latest stages of growth. Popowics et al. [10], tested the pig periosteum in three anatomical regions, in both the long-axis and transverse orientation. Although directional effect seemed mild (except for a significantly higher longitudinal peak strain in the metacarpal area), substantial differences were found in periosteal stiffness among the three bones: moduli of the zygomatic arch, metacarpal, and mandibular periosteum were 91.7 MPa, 84.7 MPa and 63.0 MPa, respectively. Mc Bride et al. [9] tested the ovine femoral diaphyseal periosteum both in an axial and circumferential orientation. The modulus of the stiff region of the stress-strain curve was 25.67 MPa, in the axial direction and 4.41 MPa in the circumferential direction. These studies also suggest that periosteal properties are influenced by life cycles [12], muscle or tendon attachments [9, 10, 12], and fibers orientation [9]. In our study, the median moduli of the steep region of the stress-strain curve was 10.75 MPa. This result was 3 times as low as in the pig mandible for a tensile test along the same axis [10]. In our study, periosteal samples were harvested in old, partially or totally edentulous subjects, in the mandibular corpus region. These facts may explain the low moduli we measured.

The results of displayed in Fig 4 confirmed that modeling the relaxation with a bi-exponential function is reasonable. This assumption was reinforced by the fact that the periosteum is composed by two materials (elastin and collagen). The two characteristic times that were measured, *τ*_1_ and *τ*_2_, were significantly less than that the duration of 24 hours between each activation during in vivo distraction (123.3, 11.6 s). Therefore, if we neglected bone remodelling, it is probable that the periosteum is in a relaxed state during a conventional distraction before each activation (one activation per 12 or 24 hours). However, it must be noted that a longer relaxation test (unavailable here) would probably show the existence of a longer characteristic time [17]. It has to be noticed that these relaxation tests were performed on cadaveric subjects aged at death of at least 75 years old, which could lead to different relaxation times than the ones in children distraction.

### Physiological state (*ε*_0_)

Like many connective tissues composed of collagen and elastin (such as skin and tendons), the periosteum is stretched on its support [8]. Therefore, to compare the stress-strain data of a tensile test with the load measured during distraction, it was necessary to determine the *ε*_0_ strain of the periosteum (its physiological state on the bone).

Despite several attempts during preliminary experiments, it was difficult to provide reliable outcomes. Indeed, the periosteum naturally shrinks when detached from its bone support, and returning the samples to their on-the-bone state required stretching along the long and short axes. Moreover, we were not able to find the exploitable retraction rates, because saline storage was responsible for excessive retraction due to tissue swelling [19].

To overcome this issue, we relied on the literature to place *ε*_0_ in the transition region of the stress-strain relationship [12, 18]. In this study, the median *ε*_0_ was 3.2%, with a corresponding median stress *σ*_0_ = 0.043 MPa. Although these values were significantly lower than the findings of other animal periosteum studies [12], but they were comparable to those of other collagen tissues [18]. Describing and modeling the behavior of connective tissues based on their microstructural organization is not new [18, 20–24]. Nevertheless, a real-time microstructural analysis in parallel with an optimized tensile protocol would allow for correlation of the organization of the ground substance (primarily collagen) with the stress and strain levels, along the elongation, and during relaxation.

### *L*_*t*_ measures

We showed an increasing load through the 20 mm distractions in this study, with a median *L*_*t*_ per millimetre ranging from 11.6 to 50.6 N. These values were of the same order of magnitude as the mean load measured in vivo by Robinson et al. (35.6 N) [25] and Burstein et al. (20 N) [26], whereas, in vivo, there is the formation of a callus, which is absent in cadavers.

### *L*_*c*_ calculation versus *L*_*t*_ measures

Using the stress-strain data of the tensile tests, we evaluated the median load applied on the distractor during a distraction to be *L*_*c*_ = 11.6 MPa. We obtained comparable data when comparing the estimated load opposing distraction generated from the tensile test *L*_*c*_ to the measured load in the first millimeter of distraction *L*_*t*_. This result confirmed that the periosteum is the primary tissue opposing distraction, which had been suggested previously without biomechanical proof.

Additionally, it is difficult to compare the aspect of the load mm curves with the J-shaped stress-strain curve of the periosteum. Indeed, considering the dimensions of an adult mandible with a 40 mm stretched periosteal segment, a 5% axial strain would correspond to only 2 mm of distraction. Moreover, provided that the mean relaxation characteristic time *τ*_2_ was 11.6 s, and that there was a mandatory latency period varying from 10 to 30 s between each activation (to save the measures and tare the torque meter), the periosteum significantly relaxed during these periods.

Although a viscoelastic model could be applied to tensile tests, it is difficult to compare beyond the first millimeter of distraction. Indeed, beyond this value the strain of the periosteum is above the maximum strain we explored in the tensile tests (see Fig 3). According to our assumptions, an activation of 2 mm would imply a strain of 20% of the periosteum in the lingual side of the mandibule. At this strain value, based on preliminary tensile tests (data not shown), some periosteum specimens ruptured. To modelize cadaveric distraction during the whole distraction process, we should assume a periosteum strain up to 200% (20 mm-distraction). Then, three issues appeared: (1) behavior of sharpey fibers out of the lifted area; (2) periosteum partial rupture; (3) relaxation between each activation. (1) In distraction, sharpey fibers are altered during the periosteum lifting and we considered that out of this area, these fibers are attached to the bone. However during 20 mm distraction, shear forces exerted on sharpey fibers could lead to their rupture. Thus, with this protocol, the periosteum slides on the bone out of the lifted area. Then the periosteum stretched area is bigger than the area considered in our calculation. (2) During preliminary experimentations, we showed periosteum rupture around 25% strain. A partial rupture of the periosteum should be considered during 200% strain. A total rupture is unlikely, since we showed a stiffening of the load during cadaver distraction. (3) To modelize distraction, the relaxation of the periosteum should be taken into account to determine the new initial state before each activation of 1 mm.

## Conclusions

This study provided the first features to understand the biomechanical role of the periosteum during distraction. We showed that the periosteum is directly stretch by the distraction activation and that the periosteum is the principal contributor to the stress involved in a distraction at the beginning of activation. Further studies should be performed to understand the origin of the stress involve in a distraction beyond the first millimeters. While the conclusion of our study seems clear, these results could be empowered and deepen by increasing the number of cadavers. The loads of distraction measured on cadaver will be useful for the development of new types of distractor devices. Sizing of activators (motorized or magnetic) depends directly on the stress involved in the distraction. Further studies with an increased number of samples, an optimized experimental method such as microstructure recording during distraction, and a microstructure-based model are required to propose a viscoelastic model of the periosteum based on this microstructure. *In fine*, the objective will be to predict the stress involved during *in vivo* from data obtained from the microstructural analysis of periosteum biopsies.

## Supporting information

S1 Text Complementary information on the distractor calibration method. (PDF)

S1 Fig Scheme of the calibration custom-made apparatus. The distractor is fixed at an immobile frame. The distractor mobile plate is attached to weights ranging from 0 kg to 6 kg. We measure the average torque required to depart the plates from 0 to 20 mm with a torquemeter.
(EPS)

S2 Fig A. Cadaver distraction (1) An horizontal incision is performed throughout the mucosa and periosteum. (2) The periosteum is lifted in the vestibular part (lateral part of the mandible) and in the lingual part (interior part of the mandible). An osteotomy is performed subperiosteally, and the distractor device is place on the bone. (3) the periosteum is sutured. (4). The activation of the distraction is performed and stretched the periosteum. B. Schema of the vestibular (lateral) sample harvesting. The sample size are drawn, the larger (40 × 10 mm) for traction test, and small sample (5 × 10 mm) for histologic assessment. Fiber collagen are represented by the pink oscillation, showing the fiber direction.
(PDF)

S3 Fig Calibration curve relying the load exerted between the two plates and the torque required to depart the plates from 0 to 20 mm. (PDF)
