# Supplementary material for: Contribution of the periosteum to mandibular distraction
Source: PLoS One. 2018 Jun 28;13(6):e0199116. doi: 10.1371/journal.pone.0199116 (PMC6023199; doi:10.1371/journal.pone.0199116)
Supplement: S1 Text — (PDF) [file pone.0199116.s001.pdf]

Supporting information

S1 Text. Materials and Methods For a given load on the distractor the torque an operator must apply on the activation rod depends on the screw-thread size and on the friction in the mechanical parts. The relationship between the load L and the torque Γ is given by the equation :

$$\Gamma = \frac{\eta L}{\pi} + \Gamma_{fric} \tag{1}$$

where η is the screw-thread size of the endless screw of the distractor (typically 0.1 - 0.5 -1.0 mm/turn in a commercial device) and Γ_{fric} is the additional torque coming from the friction into the device.

The torque which is coming from the friction is difficult to evaluation analytically. This friction comes from rotation of the endless screw into the tapped hole fixed to the moving plate, but also in the activation rod and all moving parts of the distractor. This friction can change from one device to another depending on the manufacturing quality, model and brand.

Therefore, to evaluate the friction we have designed an apparatus reported in S1 Fig. The principle is very simple. The device is fixed horizontally to an immobile frame, the moving plate is attached a weight by a thread through a pulley. Then, we adapted weights from 0 kg to 6 kg in order to apply load ranging from 0 N to 60 N approximatively and we measure the average torque to depart the two plates from 0 to 10 mm ...

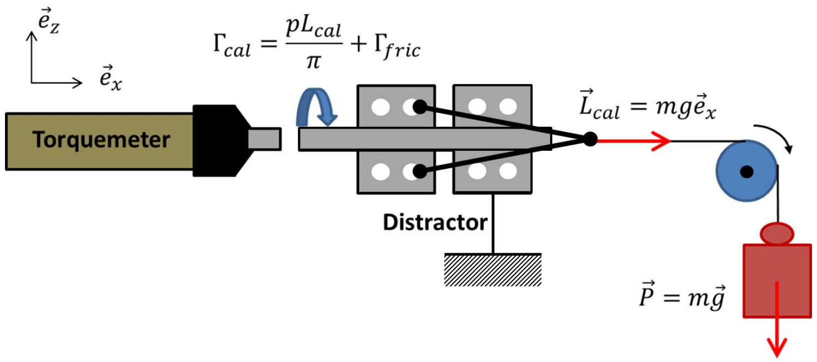

S1 Fig. Scheme of the calibration custom-made apparatus. The distractor is fixed at an immobile frame. The distractor mobile plate is attached to weights ranging from 0 kg to 6 kg. We measure the average torque required to depart the plates from 0 to 20 mm with a torquemeter.

A typical measurement for a given distraction is reported in S3 Fig. We observe that the torque required to depart the plates increases significantly above load values of 40 N. It is region where the non linear term of the friction becomes predominant. However, in our cadaver measurements, typical loads involved in distraction are between 10 and 50 N so mainly in the linear part. The calibration curves is available for every distraction device implanted, and therefor were used to evaluation the distraction force involved in every distraction performed.
