# Supplementary material for: Contribution of the periosteum to mandibular distraction
Source: PLoS One. 2018 Jun 28;13(6):e0199116. doi: 10.1371/journal.pone.0199116 (PMC6023199; doi:10.1371/journal.pone.0199116)
Supplement: S2 Fig — A. Cadaver distraction (1) An horizontal incision is performed throughout the mucosa and periosteum. (2) The periosteum is lifted in the vestibular part (lateral part of the mandible) and in the lingual part (interior part of the mandible). An osteotomy is performed subperiosteally, and the distractor device is place on the bone. (3) the periosteum is sutured. (4). The activation of the distraction is performed and stretched the periosteum. B. Schema of the vestibular (lateral) sample harvesting. The sample size are drawn, the larger (40 × 10 mm) for traction test, and small sample (5 × 10 mm) for histologic assessment. Fiber collagen are represented by the pink oscillation, showing the fiber direction. (PDF) [file pone.0199116.s003.pdf]

Supporting information

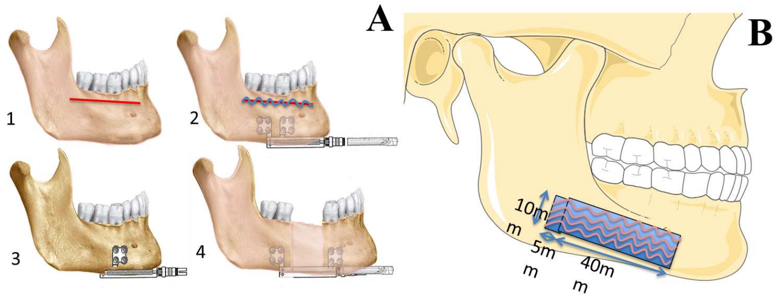

S2 Fig. A. Cadaver distraction (1) An horizontal incision is performed throughout the mucosa and periosteum. (2) The periosteum is lifted in the vestibular part (lateral part of the mandible) and in the lingual part (interior part of the mandible). An osteotomy is performed subperiosteally, and the distractor device is place on the bone. (3) the periosteum is sutured. (4). The activation of the distraction is performed and stretched the periosteum. B. Schema of the vestibular (lateral) sample harvesting. The sample size are drawn, the larger (40×10 mm) for traction test, and small sample (5×10 mm) for histologic assessment. Fiber collagen are represented by the pink oscillation, showing the fiber direction
