# Supplementary material for: Contribution of the periosteum to mandibular distraction
Source: PLoS One. 2018 Jun 28;13(6):e0199116. doi: 10.1371/journal.pone.0199116 (PMC6023199; doi:10.1371/journal.pone.0199116)
Supplement: S3 Fig — (PDF) [file pone.0199116.s004.pdf]

Supporting information

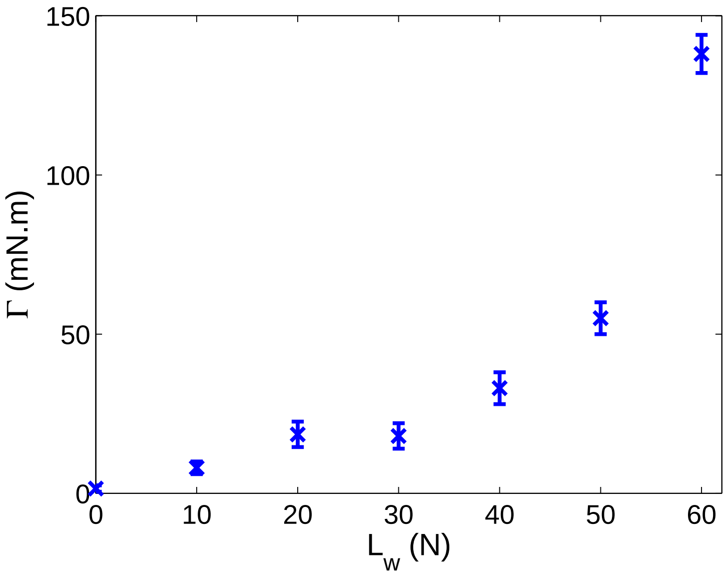

S3 Fig. Calibration curve relying the Load exerted between the two plates and the torque required to depart the plates from 0 to 20 mm.
